# An interpretable decision-support model for breast cancer diagnosis using histopathology images

**DOI:** 10.1016/j.jpi.2023.100319

**Published:** 2023-06-13

**Authors:** Sruthi Krishna, S.S. Suganthi, Arnav Bhavsar, Jyotsna Yesodharan, Shivsubramani Krishnamoorthy

**Affiliations:** a Center for Wireless Networks & Applications (WNA), Amrita Vishwa Vidyapeetham, Amritapuri, India; b Valeo, Navalur, Chennai, India; c School of Computing and Electrical Engineering, IIT Mandi, Himachal Pradesh, India; d Department of Pathology, Amrita Institute of Medical Science, Kochi, India; e Department of Computer Science and Engineering, Amrita Vishwa Vidyapeetham, Amritapuri, India

**Keywords:** Attention branch network, Breast cancer, Computer-aided diagnosis, Convolutional neural networks, Histopathology images

## Abstract

Microscopic examination of biopsy tissue slides is perceived as the gold-standard methodology for the confirmation of presence of cancer cells. Manual analysis of an overwhelming inflow of tissue slides is highly susceptible to misreading of tissue slides by pathologists. A computerized framework for histopathology image analysis is conceived as a diagnostic tool that greatly benefits pathologists, augmenting definitive diagnosis of cancer. Convolutional Neural Network (CNN) turned out to be the most adaptable and effective technique in the detection of abnormal pathologic histology. Despite their high sensitivity and predictive power, clinical translation is constrained by a lack of intelligible insights into the prediction. A computer-aided system that can offer a definitive diagnosis and interpretability is therefore highly desirable. Conventional visual explanatory techniques, Class Activation Mapping (CAM), combined with CNN models offers interpretable decision making. The major challenge in CAM is, it cannot be optimized to create the best visualization map. CAM also decreases the performance of the CNN models.

To address this challenge, we introduce a novel interpretable decision-support model using CNN with a trainable attention mechanism using response-based feed-forward visual explanation. We introduce a variant of DarkNet19 CNN model for the classification of histopathology images. In order to achieve visual interpretation as well as boost the performance of the DarkNet19 model, an attention branch is integrated with DarkNet19 network forming Attention Branch Network (ABN). The attention branch uses a convolution layer of DarkNet19 and Global Average Pooling (GAP) to model the context of the visual features and generate a heatmap to identify the region of interest. Finally, the perception branch is constituted using a fully connected layer to classify images.

We trained and validated our model using more than 7000 breast cancer biopsy slide images from an openly available dataset and achieved 98.7% accuracy in the binary classification of histopathology images. The observations substantiated the enhanced clinical interpretability of the DarkNet19 CNN model, supervened by the attention branch, besides delivering a 3%–4% performance boost of the baseline model. The cancer regions highlighted by the proposed model correlate well with the findings of an expert pathologist.

The coalesced approach of unifying attention branch with the CNN model capacitates pathologists with augmented diagnostic interpretability of histological images with no detriment to state-of-art performance. The model’s proficiency in pinpointing the region of interest is an added bonus that can lead to accurate clinical translation of deep learning models that underscore clinical decision support.

## Introduction

Breast cancer is one of the most lethal diseases in women, and its prevalence is increasing at an alarming rate. In India, 1 78 361 new cases and 90 408 deaths were reported for breast cancer in the year 2020 and it is estimated that the incidence may rise to 2 50 000 by 2030.^1^^,^^2^ Breast cancer has ranked number one cancer among Indian females with age adjusted rate as high as 25.8 per 100 000 women and mortality 12.7 per 100 000 women.^3^ This indicates the necessity of mass screening of breast health to reduce the mortality rates in India. The mortality-to-incidence ratio was found to be as high as 66 in rural registries, whereas as low as 8 in urban registries.^3^ The consequent mortality rates are notably high in developing countries, supervened by inadequate outfitted facilities for timely diagnosis and treatment. Although numerous imaging modalities aiding the diagnosis of cancer are available,^4^ the affirmative validation of cancer diagnosis and treatment is deferred to biopsied results. Histopathological assessment of biopsies is performed using microscopic examination of tissue sections stained by Hematoxylin and Eosin (H&E) and by immunohistochemical techniques. Due to the increased incidence rate of cancer in India, each pathology laboratory receives around 1000 specimens daily for evaluation.^5^ Around 50 slides will be prepared from each specimen for definitive diagnosis. These slides may consist of margins, tumor regions, adjacent areas of tumor regions, or any other relevant tissue areas. It is important for pathologists to examine all these slides to ensure a comprehensive understanding of the tumor and surrounding tissue to confirm the presence or absence of cancer and make a conclusive diagnosis. One biopsy slide itself will have hundreds of Field-of Views (FOVs), and pathologists need to carefully evaluate each FOV to make an accurate diagnosis. In the process of microscopic examination of breast tissues to distinguish benign and malignant tumors, pathologists often switch between different magnifications to focus on different tissue features, such as the overall structure, cellular morphology, and nuclear details.^6^ All these processes take a few minutes to hours to make a clinical decision on 1 biopsy slide, depending on the complexity of the cases. For difficult and borderline cases, the final histopathology diagnosis requires inputs from clinical, radiological, and other laboratory findings. In such cases, pathologist can report a final diagnosis by analyzing the correlation between the radiology and pathology findings. There may be discordant results where a lesion that is radiologically benign turned out to be malignancy by pathology or vice versa, requiring critical attention. All these factors increase the pathology workload due to the shortage of expert pathology workforce (mean number of pathologists per million population is 14)^7^^,^^8^

The tissue analysis and its diagnosis depend on the expert skill and experience of the pathologist, protocols followed in each laboratory, instruments used, staining procedures, and the approaches used to analyze the histopathological images. Of these, pathologist-related factors may be the most significant contributors to diagnostic variability.^9^ There may be subjective variations in the interpretation of each pathologist, especially in difficult borderline cases. For example, pathologists might misinterpret atypical cells with reactive cytomorphological variations as malignant tumors leading to false positives in the diagnosis.^10^^,^^11^ The misclassification of breast tumors may contribute to the over- and undertreatment of tumors. False-positive results expose patients to unnecessary costs and toxicities, whereas false-negative results may deny effective therapy to the patients.^12^ It is reported that the false-positive rate in the needle core biopsy is 0%–7.1%.^13^ Thus, it is strongly advised to use an automated system that can accurately detect breast cancer in all magnification factors to help pathologists provide timely, error-free results. Such an automated system can assist the pathologist by marking out relevant areas in the histopathology images, enabling pathologists to focus more on the difficult borderline cases to provide an accurate diagnosis, hence alleviating the burden on pathologists.

Digital Image Analysis (DIA) offers efficient and powerful tools for characterizing histopathology images and can play an essential role in improving the accuracy and consistency of disease diagnosis. DIA can identify specific features of histopathology images, such as the number and size of cells, the presence of particular cell types, and the degree of tissue damage or inflammation. By quantifying these features, DIA can provide valuable information about the nature and severity of a disease.^14^ Currently, the medical community is keenly interested in computer-aided diagnosis of cancerous cells from histopathology images.^15^ Artificial Intelligence (AI)-driven approaches aim to create AI algorithms that can perform tasks that typically require human intelligence, such as image recognition, decision-making, and experience-based learning. AI systems can process large amounts of biopsy slides quickly and accurately, which can help pathologists identify patterns and anomalies that might not be immediately apparent to the human eye and deliver promising results in breast cancer screening by detecting the signs that doctors miss.

A significant amount of work has been reported in the medical literature, apropos of identification of cancer cases pursuant to the classification of histopathological images. Previous studies bear testimony to the advocacy of transfer learning for the classification of histopathology images for the detection of breast cancer.^16^, ^17^, ^18^, ^19^, ^20^, ^21^, ^22^, ^23^ Despite the impressive performance of CNN models in the analysis of histopathology images, ambiguity in which segment of the image contributes to a particular decision prompts a question on the confidence level of the decision. The interpretability of deep learning models, particularly Convolutional Neural Networks (CNNs), poses an arduous challenge regarding medical image diagnosis. Researchers have learned visual explanations as workarounds to decipher such challenges. Visual explanations entail a top-down approach to highlight the relevant features learned by the CNN model in the arrival of its decision. Some of the reported literature used the Gradient-based visualization technique, Grad CAM with CNN model for the breast cancer histopathology image analysis.^24^ The visual explanations created by Grad CAM (extended CAM) use the gradient information and are significantly impacted by noisy gradients. Grad CAM produces heatmaps, which are applied to a pre-trained neural network on completion of the training, and the parameters are fixed. However, Grad-CAM may yield compromised performance, as the model may become over reliant on certain image regions and ignore other essential features.^25^ Hence, adopting Grad-CAM and its variants with the CNN model cannot be considered a promising solution for histopathology image analysis. These observations called for developing trustworthy alternatives to improve the interpretability of high-risk domains such as pathology image analysis.

To address these challenges, we propose a new interpretable decision-support model for histopathology image classification by leveraging transfer learning and trainable response-based attention mechanism. The model combines an attention mechanism with CNN feature extractor offers diagnostic interpretability and boosts the classification performance of histopathology images, establishing a promising approach for clinical decision-making.

Our major contributions are summarized as follows:

• A new decision-support model marshalling a DarkNet19 variant as a feature extractor for the precise inference of discriminative features for the classification of breast histopathology images. We adopted DarkNet19 variant as our base model because of its lesser depth and complexity.
• Along with the classification, we offer a visual explanation without affecting the performance of the DarkNet19 variant model; the integration of the visualization techniques increased the classification accuracy. This was accomplished by fusing the attention branch with the DarkNet19 model to create the Attention Branch Network (ABN), which provides diagnostic interpretability for a thorough analysis of pathologic histology. Substantiated the conspicuous efficacy of the ABN-DarkNet19 variant network in preference to the baseline DarkNet19 variant network.
• Demonstrate the propriety of attention map-based explanation of classification results, by emphatic visualization, of cancerous regions in the images. We obtained CAM visualization of the DarkNet19 variant model and compared it with the visual explanation by the ABN-DarkNet19 variant model. We submit that the ABN-DarkNet19 variant model locates the cancer regions which closely match with pathologist's findings. ABN-DarkNet19 variant model provides better diagnostic interpretability, which inherently helps to improve the pathologist’s trust in automated AI systems. To the best of our knowledge, we are the first to provide an explainable comparison of CAM and ABN visualization techniques using breast histopathology image datasets.

## Related works

This section discusses the current works reported in the field of histopathology image analysis. The availability of high spatial resolution digitized histopathology image data, and progressive advancements in the domain of AI have stimulated fervent interests in recognizing abnormality in histopathological images.^26^ Mastrosimini et al^27^ reported review on validation of Whole Slide Imaging (WSI), and validated the effectiveness of WSI over microscopic evaluation of glass slides. Various flavors of neural networks and conventional machine learning techniques are exploited for the recognition of malignancy from breast histopathology images.^28^, ^29^, ^30^, ^31^, ^32^

The introduction of deep learning techniques, especially Convolutional Neural Networks (CNNs), has been noticeably effective in detecting anomalies within histopathology images.^16^, ^17^, ^18^, ^19^, ^20^, ^21^, ^22^, ^23^, ^24^, ^25^, ^26^^,^^29^^,^^30^^,^^33^, ^34^, ^35^, ^36^ Recent advances in the application of CNN and the introduction of the transfer learning technique offered a propitious solution to the performance of CNN, even with small-sized histopathology image datasets. In transfer learning, a network trained on one domain is fine-tuned and deployed to serve an application from another domain. Transfer learning via CNN models trained on the large-scale ImageNet dataset is widely adaptable for classifying histopathological images.^37^, ^38^, ^39^, ^40^ The work in^16^, ^17^, ^18^, ^19^, ^20^, ^21^, ^22^, ^23^, ^24^ employed transfer learning techniques using existing deep neural networks such as AlexNet, VGGNet, InceptionNet, ResNet, etc, that are trained using ImageNet dataset. Sun et al meticulously scrutinized the efficacy of the transfer learning technique, training CNN from scratch, reaffirming that transfer learning is better effective.^18^

Togacar et al developed the BreastNet model, which consists of a convolutional block attention module (CBAM), dense block, residual block, and hypercolumn technique.^19^ Benhammou et al and Zhou et al presented a comprehensive review of the CNN models designed for BreaKHis image analysis.^20^^,^^33^ Xie et al pursued supervised and unsupervised Deep Convolutional Neural Networks (DCNN) for the categorization of breast cancer using images from BreaKHis dataset.^21^ They adopted Inception v3 and Inception ResNet v2 architectures using transfer learning techniques. Liew et al presented DenseNet21 with XGBoost classifier to analyze breast histopathology images. They performed binary and 8-class classifications of images using the proposed model.^22^

Gupta et al presented a sequential framework that uses multi-layered deep features derived from DenseNet.^37^ Dimensionality reduction was performed using the XGBoost technique. Boumaraf et al investigated a new transfer learning method based on block-wise fine-tuning strategy to capture the best intrinsic features from the histopathology images. The model is validated using magnification dependent/independent dataset for both binary and 8-class classification.^23^ Gour et al^41^ proposed a ResHist CNN model for breast cancer histopathology image classification. The images are pre-processed using stain normalization, data augmentation, and patch extraction. Abdulla et al reviewed breast cancer classification using CNN, Long-Short-Term-Memory (LSTM) approach, and a combination of the 2 as feature extractors. Softmax and Support Vector Machine (SVM) classifiers were used for the classification.^42^ Thuy et al proposed a unified network combining deep learning, transfer learning, and generative adversarial network (GAN) to enhance the classification performance.^43^ They used the VGG16 network and then both VGG16 & VGG19 for feature extraction. Additionally, they highlighted that GAN implementation reduced the performance of their CNN models.

Recently, researchers are keenly interested to implement CNN models with attention mechanisms to visually analyze the prediction of CNN models and provide region-level supervision to spot errors and uncertainty in the network's decisions.^44^^,^^45^ Yang et al introduced the Guided Soft Attention Network for the classification of histopathology images towards the detection of breast cancer.^46^ BenTaieb et al presented a recurrent visual attention model with inception network capable of attending to the discriminative regions of an image.^47^ Tomita et al elucidated grid-based attention mechanism with ResNet18 model for high-resolution microscopy image analysis.^48^ Yao et al proposed Deep Attention Multiple Instance Survival Learning by introducing both Siamese networks based on the attention model, and a fully connected layer, for histopathology image analysis, solve the challenge of less annotated patch images.^49^ Huang et al proposed an end-to-end Depth Domain Adaptive (DDA) Network with integration gradient CAM and a priori experience-guided attention to improve the tumor grading performance and interpretability by introducing the pathologist's a priori experience in high magnification into the depth model using low magnification.^6^ Huang et al proposed another novel fusion attention block network (FABNet) for laryngeal cancer tumor (LCT) grading.^50^ Subramanyam et al presented Local Residual Attention Network (LRAN) for breast histopathology image analysis.^51^ The authors claimed that the proposed LRAN improved the performance of baseline CNN models. However, the visualization of tumor regions reported in these works are not correctly consilient with pathologist’s findings. The credibility of these models is yet to be evaluated to make them suitable for clinical use.

Grad CAM-based visualization technique is explored for histopathology image analysis.^52^^,^^53^ However, the area of interest in the images located by Grad CAM is not correctly aligned with the tumor regions. It is reported that Grad CAM produces heatmaps applied to an already-trained neural network after training is complete, and the parameters are fixed.^54^ Grad CAM does not provide an overall explanation of the model,^55^ simply highlights the areas of the image that contribute most to the classification.^56^ In some cases, Grad CAM visualization may even reduce performance as the model may become over-reliant on certain regions of the image and not take into account other important features.

Class Activation Mapping (CAM) is also utilized to highlight the region of interest where the CNN model focuses for decision-making for histopathology image analysis.^57^ CAM is a response-based visual explanation technique that uses extracted feature maps from the inference layers to interpret the decision-making process of a CNN model.^58^ CAM processes response values in a convolutional layer and the connection weights of a fully connected layer.^59^ Since CAM requires a Global Average Pooling (GAP) layer with a convolution layer of interest, it makes it computationally expensive. CAM replaces the final fully connected layer of the network with GAP and convolutional layer, degrading the performance of the model.^58^ To address these challenges, response-based trainable attention mechanisms are introduced to visualize regions of interest in the CNN model's decisions.^25^ Attention Branch Network (ABN), based on trainable attention mechanism, becomes popular to visualize the CNN network's decision.^60^ ABN is exploited to enhance the classification performance of CNN models and highlights regions of interest on various computer vision tasks.^61^^,^^62^ The potential of ABN is yet to be explored for the classification of breast histopathology images.

In this paper, we propose a new decision-support model that utilizes the potential of ABN to enhance the performance of a CNN model for the analysis of pathologic histology. The proposed model not only aims to improve the classification accuracy, but also aims to provide diagnostic interpretability, enabling the model to explain the reasoning behind its decisions.

## Proposed methodology

**Fig. 1:**
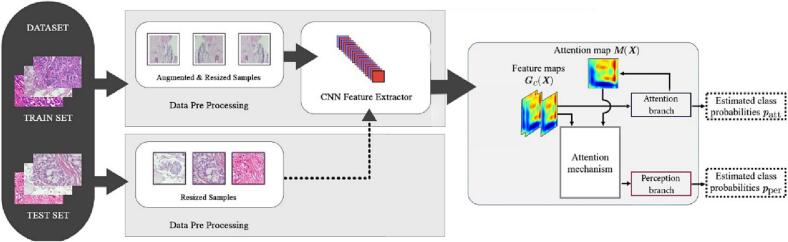
Overall flow diagram of the proposed model. Images from the dataset are split into the training set and testing set. Pre-processed images are fed to the proposed model. The model consists of a Feature extractor, an Attention branch, and a Perception branch. This model has DarkNet19 variant as a feature extractor. The extracted feature maps are given to the attention branch to generate the attention map. The extracted feature map and attention map are fed to the perception branch for binary classification of breast histopathology images.

This section elucidates the suggested interpretable decision-support model for the precise classification of breast cancer histopathological images. Fig. 1 illustrates a flow diagram of the proffered approach.

In Fig. 1, the images in the dataset are split into training set and testing set. The discriminative features from the input images are extracted using a CNN feature extractor. We adopted DarkNet19 network variant, DarkCovidNet (DCN) reported in Ozturk et al,^63^ as the base model. DCN model is selected as a feature extractor because of the provision of lesser depth with fewer parameters without compromising performance accuracy. We applied transfer learning on the DCN model and fine-tuned it for BreaKHis histopathology images. In order to boost the performance of DCN model, an Attention Branch Network (ABN) that combines the attention branch with DCN feature extractor is introduced. This unified unit, referred to as the ABN-DCN model, upholds an unambiguous classification of histopathological images; besides, the concurrent visualization of cancerous regions promotes diagnostic interpretability. The perception branch classifies images into benign versus malignant classes.

### Dataset description

The proposed decision-support model was assessed using BreaKHis, a publicly accessible dataset from P & D Lab, Brazil.^39^ An Olympus BX-50 system microscope with a relay lens with magnification of 3.3× coupled to a Samsung digital color camera SCC-131AN is used to obtain digitized images from the breast tissue slides. This camera uses a 1/3” Sony Super-HAD (Hole-Accumulation Diode) interline transfer charge-coupled device with a 6.5 μm×6.25 μm pixel size. The BreakHis dataset obtained from 82 patients, was comprised of 7909 images with a size of 700×460×3. The images constituted of 2480 benign and 5429 malignant samples. The dataset also comprised of diverse levels of image magnifications of 40×, 100×, 200×, and 400×, corresponding to objective lens 4×, 10×, 20×, and 40×. The adequate pixel size in the object plane is 0.49 μm at 40×, 0.20 μm at 100×, 0.10 μm at 200×, and 0.05 μm at 400×. The benign subset of images in the dataset was categorized into the following classes: Adenosis (A), Fibroadenoma (F), Phyllodes Tumor (PT), and Tubular Adenoma (TA). Malignant classes were designated as follows: Ductal Carcinoma (DC), Lobular Carcinoma (LC), Mucinous Carcinoma (MC), and Papillary Carcinoma (PC). This paper’s investigation focused on binary classification, generalizing the precedent categories into benign and malignant classes. The performance of the ABN-DCN model was analyzed using images of 40×, 100×, 200×, and 400× magnification factors with respective sizes of 1995, 2081, 2013, and 1820 samples. In order to eliminate overfitting, data-augmentation techniques like rotations 25 °C and horizontal flips were applied to beef up the number of images in training set for all classes**.**

### Interpretable decision support ABN-DCN model

**Fig. 2:**
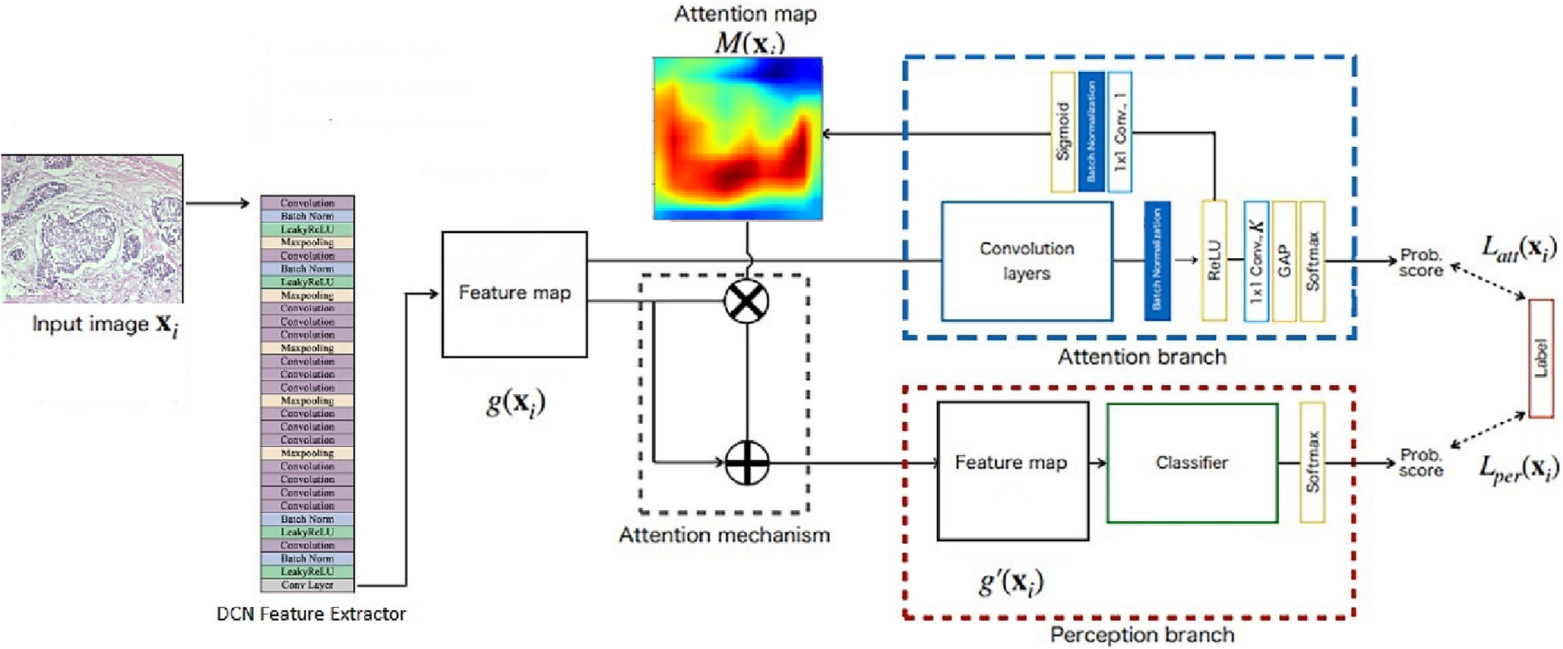
Architecture of proposed decision-support ABN-DCN model. DCN Feature extractor with 17 DarkNets and 5 max pooling layers is used to extract relevant feature maps. The attention branch is integrated with the DCN feature extractor to generate attention maps using attention mechanisms. The attention maps and feature maps are fed to the perception branch to classify the histopathology images.

Fig. 2 depicts the architecture of proposed ABN-DCN model.

The ABN-DCN model is an attention branch network that consists of a feature extractor, an attention branch, and a perception branch. The feature extractor extracts relevant feature maps from BreaKHis dataset images. Interpretability of the CNN model is facilitated by the collaborated interplay of the attention branch and the feature extractor modules. The attention branch generates attention maps by using the feature maps from the DCN feature extractor. The perception branch’s main function is to process the received features and attention maps to infer the probability of each class.

#### Overview of ABN-DCN feature extractor

We adopted the DCN model, a variant of DarkNet19, as the feature extractor for ABN-DCN model. The baseline DCN model employs a smaller number of convolutional filters than DarkNet19 without compromising the performance of the model. This markedly simplifies the complexity and training time of the model. The DCN model was specifically developed and fine-tuned to detect COVID-19 from chest X-ray images.^63^ The potential of baseline DCN model has not yet been utilized for breast histopathological image analysis.

The DCN feature extractor block shown in Fig. 2 is comprised of 17 convolution layers, where the Batch Normalization layer and Leaky ReLU operations follow each layer. Each set of 3 consecutive convolution layers has identical configurations 3 times successively. The batch normalization operation was embraced for the reduction of training time and enhancement of the model’s stability. Similar to the DarkNet19, DCN model adopts Max Pooling to downsize an input by considering the maximum of a region selected by its filter. The image resolution is modified to 256×256 pixels to suit the requirement of input image sizes, conforming to the mentioned DCN model.^63^ The feature maps from the final convolution block of DCN model are fed to the attention branch to generate attention maps to provide diagnostic interpretability.

#### Attention branch network (ABN)

ABN is the primary method for performance improvements of CNNs, prompted by the extension of an attention map to the attention mechanism in feed-forward propagation for visual explanation. The coalition of a branch structure and an attention mechanism enabled the extension of a response-based visual explanation in the submitted decision-support model. The attention branch is constructed after feature extractor on the basis of the Class Activation Maping (CAM). CAM, with K=2 classes, consists of K×3×3 convolution layer, global average pooling (GAP), and a fully connected layer.^60^ K×3×3 convolution layer extracts a K×h×w feature map that highlights the gaze area in a particular class. CAM performs global average pooling (GAP) on the convolutional feature maps and down samples the feature maps to K×1×1. After training, CAM generates an attention map by manipulating the feature map and weight at the fully connected softmax layer. CAM is unable to generate an attention map while being trained. Therefore, CAM introduced 3×3 convolution layer and get rid of the fully connected layer. However, the introduction of 3×3 convolution layer to maintain the spatial information contained in the output of the last convolution layer decreases the performance of CNN. To address this challenge, ABN adopts a K×1×1 convolution layer in preference to the fully connected layer, serving as the final fully connected layer of CAM in feed-forward computations.^60^

Following the K×1×1 convolution layer, the attention branch of ABN outputs the probability values of the class using GAP and the softmax function. The attention branch additionally generates attention maps from K×h×w feature maps. These K×h×w feature maps are convolved by 1×1×1 convolutional layer to generate 1×h×w feature maps, which are used to aggregate K feature maps. The 1×h×w feature map is then normalized using the sigmoid function so that it can be utilized as an attention map for the attention mechanism.

On acquisition of the attention and feature maps, the perception branch outputs the final probability for each class. The attention map is applied to the feature map by an attention mechanism. Eq. (1) explains the attention mechanism used in the ABN.

(1) $$g_{c}^{\prime }\left(x_{i}\right)=\left(1+M\left(x_{i}\right)\right).g_{c}\;\left(x_{i}\right)$$

Here, g_c_′(x_i_) is the feature map at the feature extractor; M(x_i_) is an attention map; and g_c_′(x_i_) is the output of the attention mechanism, where c |1, …, C is the index of the channel. Eq. (1) highlights the feature map in the area where the attention map strongly responds and prevents the feature map from disappearing even in the zero region of the attention map.

In this paper, the BreaKHis dataset was trained by the selection of the training loss of attention branch and perception branch using softmax function and cross-entropy. The DCN feature extractor is optimized by receiving the gradients of the attention and perception branches. ABN can be trained in an end-to-end manner, using losses at both attention and perception branches. The training loss function L(x_i_) is a simple sum of losses at both branches, as expressed by Eq. (2). Here, L_att_(x_i_) denotes training loss of input image x_i_ at the attention branch, and L_per_(x_i_) denotes training loss at the perception branch.

(2) $$L\left(x_{i}\right)=L_{\mathrm{att}}\left(x_{i}\right)+L_{\mathrm{per}}\left(x_{i}\right)\;$$

## Experimental results and discussion

This section discusses the empirical assessment based on experiments with the proposed interpretable decision-support ABN-DCN model for breast histopathological image classification**.**

### Experimental setup for model training

We used a magnification-dependent BreaKHis dataset where we trained 40×, 100×, 200×, and 400× magnified images separately. All input images are resized into 256×256 pixels concomitant with the baseline DCN model. The dataset was split into independent training and validation sets with a train test split of 70%–30%. An instance of the Tesla K-80 GPU provided by Google Collaboratory was availed for the experimental verification of ABN-DCN model. Google Colaboratory is a collaborative setup of the Jupyter/python notebook editing environment,^64^ considered highly dependable for image analysis. We followed a similar training strategy for 40×, 100×, 200×, and 400× magnification factor datasets. The optimizer stochastic gradient descent, weight decay with the momentum of 0.9, and learning rate of 0.1 are selected for the model training. The ABN-DCN model has trained over 200 epochs and has a mini-batch size of 32. The proposed ABN-DCN model is evaluated in terms of different metrics: accuracy, precision, recall, and F1 score, which can be derived as shown in Eqs. (3), (4), (5), (6), In these equations, TP, TN, FP, and FN indicate true positive, false positive, true negative, and false negative, respectively. TP is the number of images correctly interpreted as malignant tumor images in the test set. TN is the number of images correctly classified as benign tumor in test set. FP is the number of images that were incorrectly classified as malignant tumor in the test set. FN is the number of images incorrectly interpreted as benign tumor in the test set.

(3) $$\mathrm{Accuracy}=\frac{\mathrm{TP}+\mathrm{TN}}{\mathrm{TP}+\mathrm{FP}+\mathrm{TN}+\mathrm{FN}}$$

(4) $$\mathrm{Precision}=\frac{\mathrm{TP}}{\mathrm{TP}+\mathrm{FP}}$$

(5) $$\mathrm{Recall}=\frac{\mathrm{TP}}{\mathrm{TP}+\mathrm{FN}}$$

(6) $$F1\;\mathrm{Score}=2\;x\frac{\mathrm{Precision}\;x\;\mathrm{Recall}}{\mathrm{Precision}+\mathrm{Recall}}$$

### Diagnostic interpretability: Attention map visualization

BreaKHis dataset comprised benign and carcinoma-malignant image samples. Carcinoma tumors are developed due to intraductal growth of epithelial cells, which make up the surface layer of many tissues in the body. Benign tumors are referred to as fibroepithelial lesions because, histologically, they consist of epithelial elements surrounded by variable amounts of stroma.

**Fig. 3:**
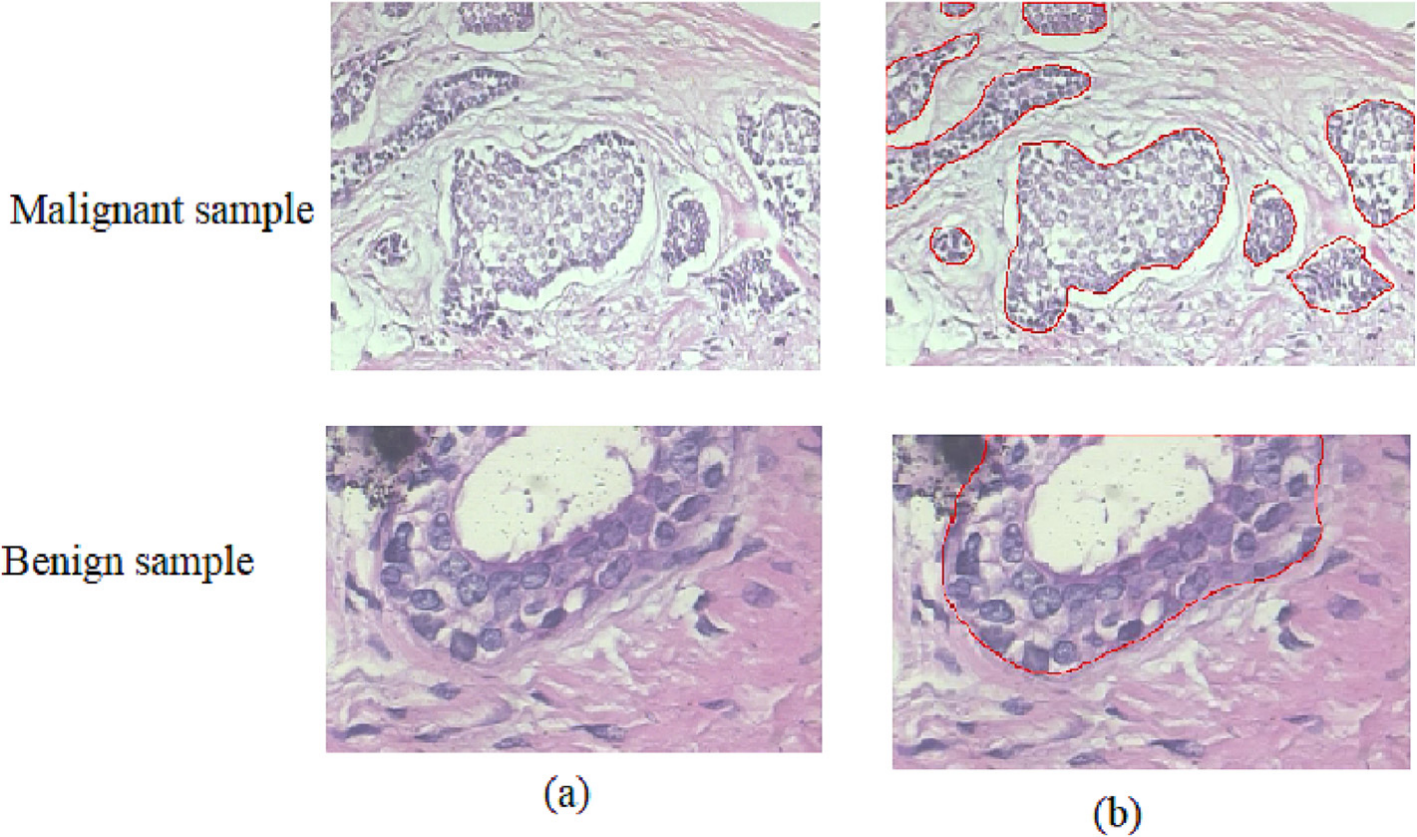
(a) Input image samples from BreaKHis dataset (b) pathologist's annotation.

Fig. 3 depicts the annotations done by collaborative pathologist at Amrita Institute of Medical Sciences (AIMS) Research Center, a super-specialist medical facility in India. The epithelial areas in the images are analyzed for annotating the abnormal areas in the benign and malignant tumors. Pathologist used QuPath software to annotate the region of interest in each pathology image.^65^

We generated attention maps for the ABN-DCN model to analyze its performance and identify the areas where it focuses for decision making. In these maps, abnormal regions in both benign and malignant tumors are highlighted. Abnormal areas are shown in red, while normal areas are shown in blue in the attention maps. The ABN-DCN model's performance is evaluated by manually verifying whether the highlighted regions in the attention maps align with the pathologists' findings. We also evaluated the diagnostic interpretability of ABN-DCN model and demonstrated its superiority over the baseline DCN model. We compared the region-level supervision of the baseline DCN model and ABN-DCN model with the pathologist's annotations to gain insights into the better performance of the ABN-DCN model.

#### CAM visualization of baseline DCN model

**Fig. 4:**
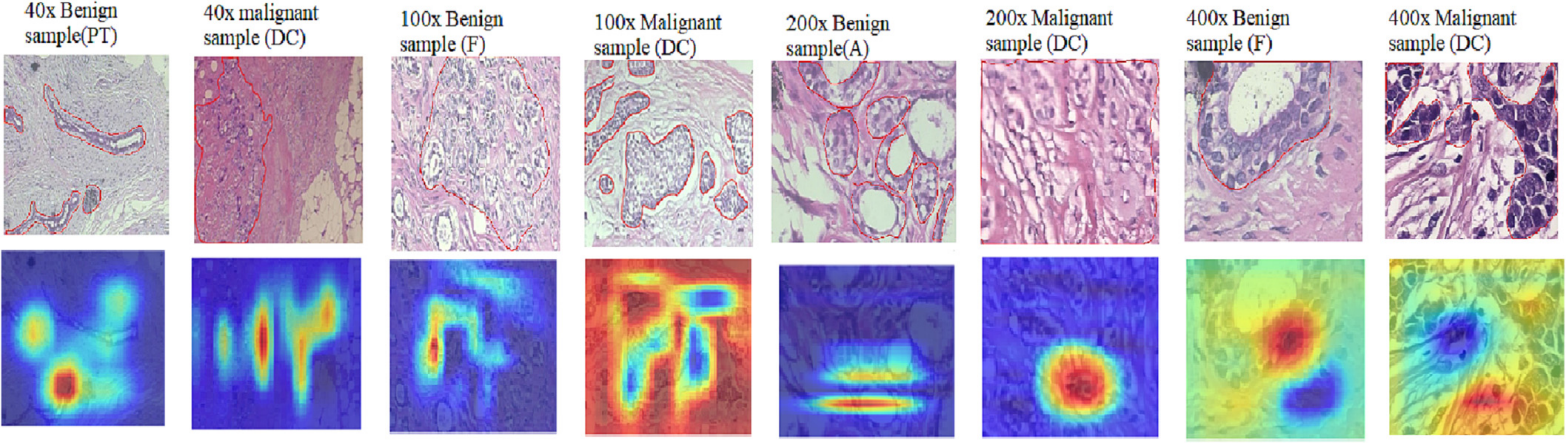
The figure shows the BreaKHis image samples with pathologist's annotation. The CAM visualization of the baseline DCN model is also depicted in the figure.

To analyze the reliability of the baseline DCN model, we generated the heatmaps using response-based CAM visualization to identify where the model focuses for decision-making. A few numbers of benign and malignant image samples with 40×, 100×, 200×, and 400× magnification factors were randomly selected from BreaKHis dataset for the visualization. The selected images were manually annotated by the collaborative pathologist. Fig. 4 depicts the heatmap visualization of the baseline DCN model.

Fig. 4 depicts adenosis, fibroadenoma, phyllode tumor benign lesions, and ductal carcinoma malignant samples. DCIS is a non-invasive, cancerous intraductal growth of epithelial cells limited to the ducts and lobules. Clinically, fibroadenoma and phyllode tumors present as movable, rounded, or lobulated masses. Both tumors are referred to as fibroepithelial lesions, as histologically, they comprise epithelial elements surrounded by variable amounts of the stroma. Adenosis is characterized by the presence of an increased number of lobules, which are the milk-producing glands in the breast. From Fig. 4, it is evident that the baseline DCN model was not able to accurately extract features from the tumor areas in the images, leading to inconsistencies with the pathologist's findings. Therefore, the baseline DCN model alone cannot be a reliable solution for breast histopathology images.

#### Attention map visualization of ABN-DCN model

**Fig. 5:**
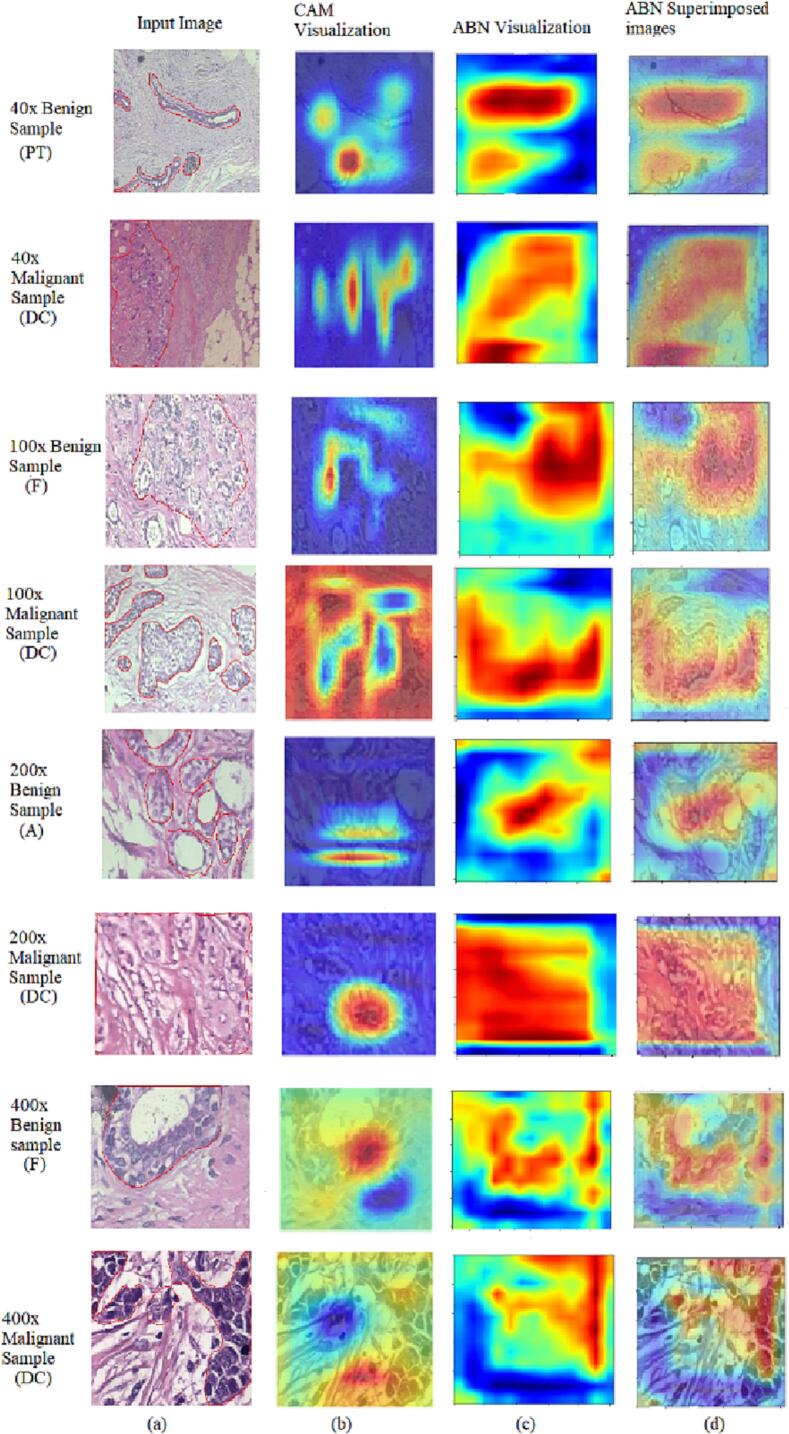
Attention map. Visualization of correctly classified histopathology images. (a) Annotated image samples with magnification factors 40×, 100×, 200×, and 400× (Top to Bottom). (b) CAM visualization of baseline DCN model. (c) Attention maps generated by ABN-DCN model (d) superimposed attention maps and input images. The pathologist may utilize the input image & attention map to finally focus the region of interest for confirming the prediction made by our model.

Fig. 5 displays the attention map generated by ABN-DCN model using images drawn from the BreakHis dataset. Comparing the attention maps generated by ABN-DCN model and CAM visualization of the baseline DCN model (Fig. 5(b) and (c)), the ABN-DCN model precisely locates the epithelial areas in the images and discriminate benign and malignant samples in the dataset. Close scrutiny of the regions highlighted by the ABN-DCN model revealed a strong correlation with the pathologists’ clinical findings and annotations. We observed that the baseline DCN model misclassified 40× malignant sample (DC), 100× benign sample (F), 200× benign sample (A), and 400× benign sample (F) in Fig. 5. ABN-DCN model correctly classified these images by locating precise tumor regions in the images.

### Performance comparison of ABN-DCN model with low footprint CNN models

Comparative appraisals of ABN-DCN model with other existing low-footprint CNN models such as baseline DCN model, VGG16, EfficientNetB0, MobileNet^63^^,^^66^, ^67^, ^68^ are tabulated in Table 1. We applied the transfer learning technique on these models and fine-tuned for histopathology images in the BreakHis dataset with magnification factors 40×, 100×, 200×, and 400×. We evaluated the classification performance of these models using popular benchmark metrics-accuracy, precision, recall, and F1 score and summarized in Table 1. The baseline DCN model outperforming conventional low-footprint CNN models bears testimony to the selection of the DCN model as a wise choice for the development of an interpretable diagnostic model. We also noticed that, integration of the attention map with the baseline DCN model forming the ABN-DCN model, greatly increased the classification performance compared to other CNN models.

**Table 1:** Performance comparison of the proposed ABN-DCN model with pre-trained deep learning models for all magnification levels of the BreaKHis dataset.

CNN model	Metric	40×	100×	200×	400×
VGG16	Accuracy	91.2	93.2	91.7	94.3
	Recall	90.0	83.5	87.4	96.8
	Precision	92.1	97.8	93.7	93.5
	F1 Score	91.2	90.1	94.0	94.0
MobileNet	Accuracy	93.7	96.4	95.9	90.1
	Recall	95.0	94.7	91.2	80.4
	Precision	93.1	91.5	96.1	90.8
	F1 Score	94.0	93.1	96.1	85.3
EfficientNet	Accuracy	94.2	95.8	94.6	94.7
	Recall	94.6	94.6	94.7	93.1
	Precision	93.3	92.8	96.2	95.5
	F1 Score	93.9	93.7	96.4	94.3
DCN model	Accuracy	95.3	96.2	96.7	93.8
	Recall	95.5	95.5	97.5	92.7
	Precision	96.0	94.5	96.0	92.5
	F1 Score	96.0	95.0	96.5	94.0
ABN-DCN model	Accuracy	98.4	98.6	98.7	97.8
	Recall	98.3	98.6	98.8	97.4
	Precision	98.4	98.5	99.1	97.8
	F1 Score	98.4	98.5	98.9	97.5

Considering the performance of CNN models using 40× magnified images in Table 1, the proposed ABN-DCN model classified benign tumors from malignant tumors with 98.4% accuracy, a noticeably improved performance over the baseline DCN model, which exhibited a classification accuracy of 95.3%. The results substantiated the propriety of integration of the attention branch with the baseline DCN model, achieving a significant improvement of 3.25% using the ABN-DCN model. Pathologists depend on low magnification factors to offer a wide field of view to examine relevant areas in tissue to understand the architectural details of the tissue. These images provide an overview of the structure and health status of the histopathological images. The recommended ABN-DCN model is capable of extracting abstract features from 40× (low-power) magnified images to distinguish malignant tumors from benign tumors.

Pathologists prefer to use (medium power) 100× and 200× magnified images to examine the detailed structure of tissues. These magnification factors provide closer views of individual cells or structures within tissue samples. Results in Table 1, clearly demonstrate that our proposed model classified benign and malignant tumors with 98.6% and 98.7% classification accuracy. ABN-DCN model achieved a marked improvement of 2.5% and 2.1% in classification accuracy for 100× and 200× magnified images.

The higher magnification factors, like 400×, usually provide the cytomorphological features of nuclei, cytoplasm, etc rather than architectural details of the tissue images. These intricate features facilitated the ABN-DCN model's capability to discriminate benign tumors from malignant tumors with 97.8% accuracy. In the case of 400× images, the ABN-DCN model achieved a significant improvement of 4.26% in accuracy compared to the baseline DCN model.

**Fig. 6:**
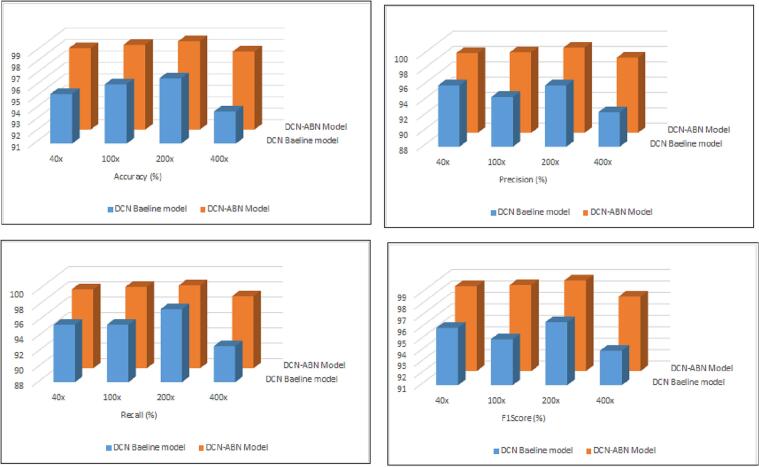
Comparison of baseline DCN model^63^ with proposed ABN-DCN model.

In conclusion, the ABN-DCN model achieved the highest accuracy on 200× magnified images, but poorer performance with 400× magnified images. The suggested ABN-DCN model was shown to effectively classify images with diverse magnification factors in the BreaKHis dataset, at both the architectural and cytomorphological levels. The unification of ABN with the DCN model overcame the weakness of the baseline DCN model, turning out a competitive benchmark performance. Fig. 6 depicts the comparative summary of performance metrics of the ABN-DCN model with the baseline DCN model.

The graphs clearly demonstrate the conspicuous improvement with the ABN-DCN model which also reduces the false-negative and false-positive rates significantly compared to the baseline DCN model. The goal at this point is to determine which magnification factor would offer a comprehensive understanding of the structure of cancerous or non-cancerous cells. Among all the magnification factors, 200× reported better classification performance compared to 40×, 100×, and 400× magnified images. Therefore, we submit that the 200× magnification factor is the most dependable one for the ABN-DCN model that can help pathologists distinguish benign tumors from malignant tumors by analyzing a closer view of the tissue structures with a clear idea of the architecture of the tissue. The ABN-DCN model significantly reduces the false positives and negatives in the prediction of BreaKHis breast histopathology images.

### Train and validation loss

There are 2 forms of losses in machine learning (ML); validation loss and training loss. Training loss interprets the extent of error incurred by the algorithm while learning from the training dataset.

**Fig. 7:**
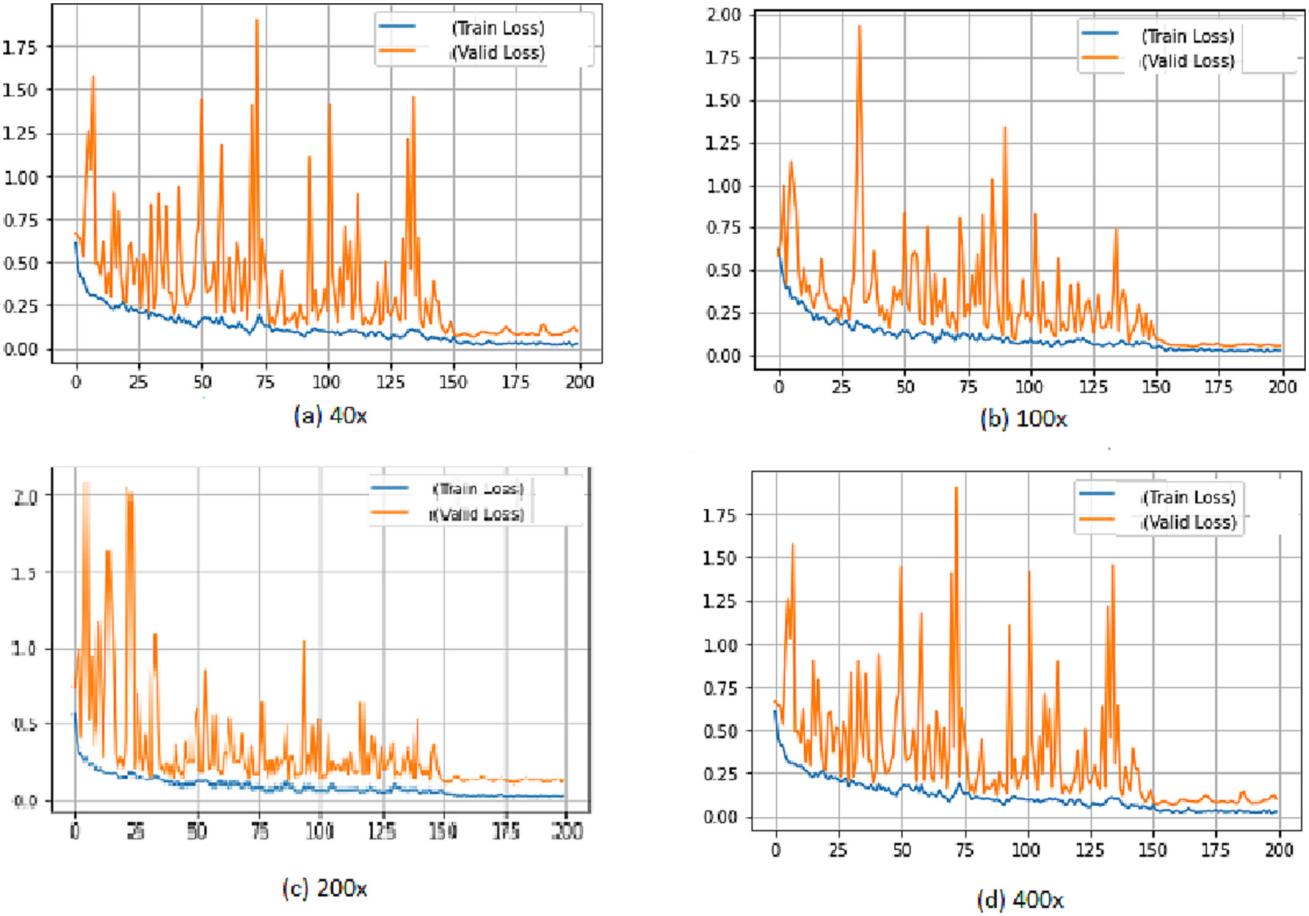
Validation loss curve of proposed CNN model (a) 40× (b) 100× (c) 200× (d) 400×.

The validation loss measures how closely the proposed model predicts labels on a new test dataset. By analyzing the shape and trends of the validation loss curve, ML practitioners can gain insights into the stability of their models, and accordingly tweak the training process, for enhanced performance and prevent overfitting. Resume Fig. 7 shows the validation loss curves produced from the submitted ABN-DCN model, subsequent to training the tissue images of 40×, 100×, 200×, and 400× magnifications. The graphs exemplify the ABN-DCN model's capability to minimize training and validation losses, indicating that the model has a little overfitting problem and is capable of generalizing new data samples with acceptable accuracy. Besides showing the least amount of variance, Fig. 7 affirmed that training with 200× images was significantly stabilized in fewer epochs in comparison to the images of other magnification levels.

### T-SNE visualization

**Fig. 8:**
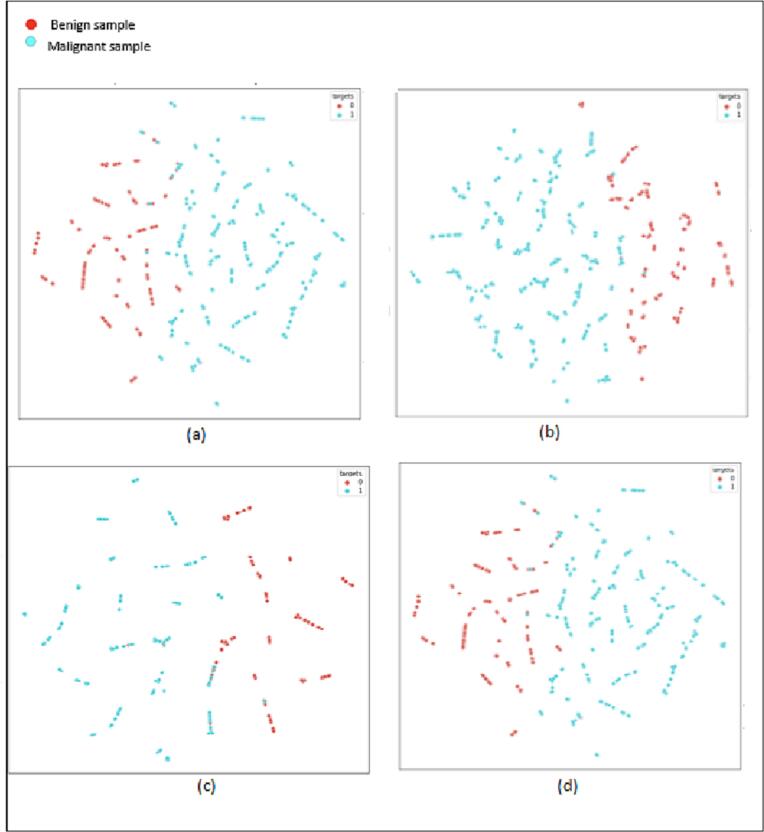
t-SNE visualization of proposed CNN model (a) 40× (b) 100× (c) 200× (d) 400×.

Sample visualization is generated by the t-Distributed Stochastic Neighbor Embedding (t-SNE) visualization scheme. t-SNE generates a 2-dimensional representation of samples that form clusters of similar points, which simplifies visual identification of the samples. Fig. 8 shows the t-SNE representation of the last layer of DCN model. The t-SNE technique visualizes a high-dimensional representation amid lower virtual dimensions. The visualizations underscore the discernible segregation of the malignant samples from benign samples. Green points are associated with malignant samples and red points represent benign images. t-SNE visualization reveals distinct characteristics of the red samples from green samples. This indicates that malignant samples are clearly distinct from benign samples as they show disparate behavior in the feature space. t-SNE was employed primarily for visualization of the behavior of feature representations. The classification was conducted in an actual feature space rather than a reduced feature space.

### Limitations of ABN-DCN model

**Fig. 9:**
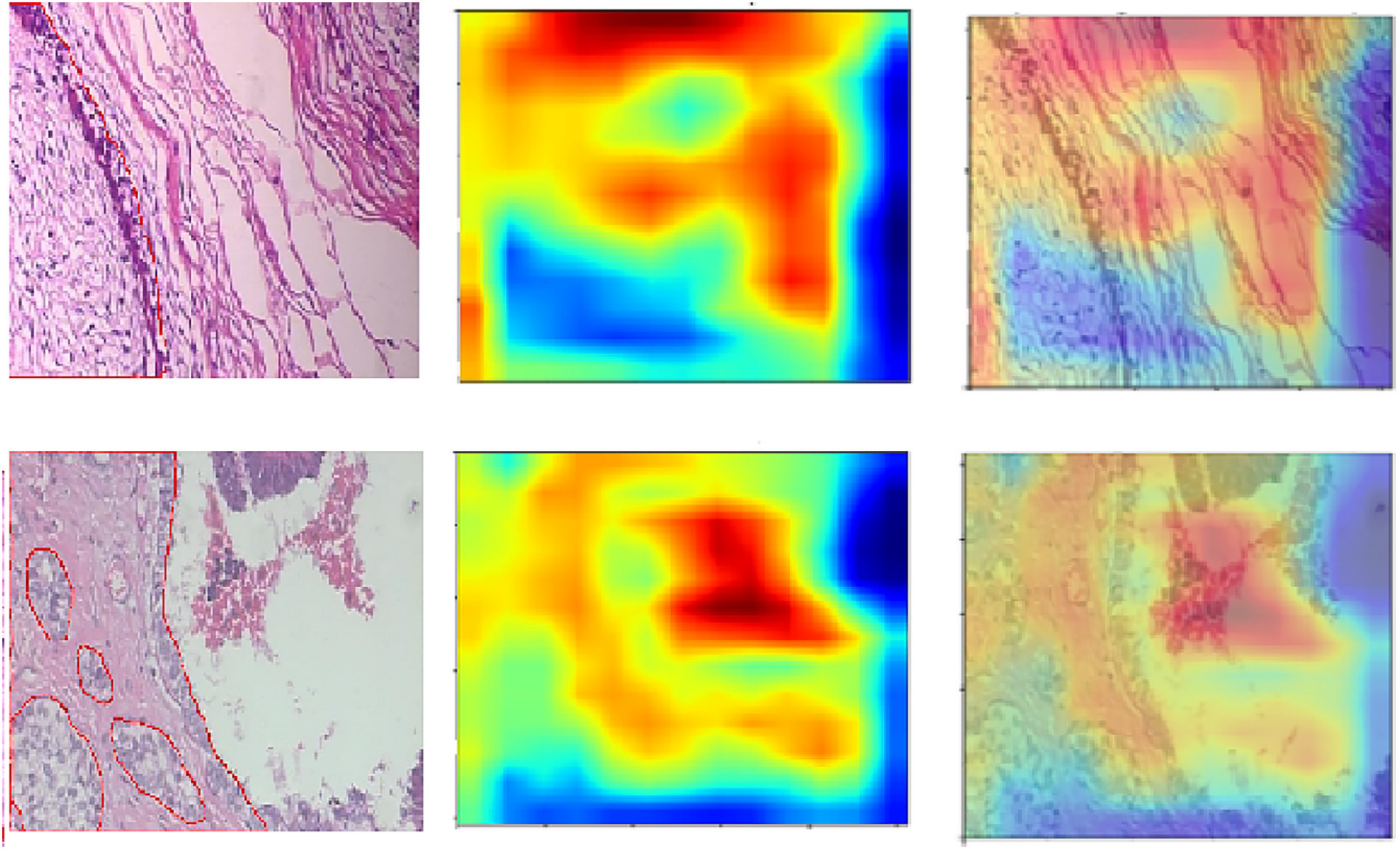
Attention map. Visualization of incorrectly classified histopathology images. (a) Input image samples, (b) attention map generated, (c) superimposed input image samples and attention maps. Visualization of these images has shown that our model incorrectly classified a marginal number of images with imprecise region identification.

From Table 1, we observed that the ABN-DCN model incorrectly classified a few images in the BreaKHis dataset. Considering the 200× image dataset, ABN-DCN model achieved a recall of 98.8%, indicating that 1.2% of benign tumors were incorrectly classified as malignant tumors. The reliability of the suggested ABN-DCN model was appraised by the generation of attention maps of incorrectly classified images, as shown in Fig. 9.

The ABN-DCN model's highlighted regions do not fully align with the pathologist's findings. However, the visual representation shows a significant overlap between the abnormal regions in the original images. This suggests that fine-tuning the ABN-DCN model can enhance its ability to classify benign and malignant tumors accurately.

Based on these findings, we submit that the recommended ABN-DCN model achieved better classification performance and interpreted cancerous regions in the histopathology image with high confidence, i.e., a promising solution for efficient classification of histopathology images with diagnostic interpretability.

We trained and validated our proposed model using BreaKHis dataset which primarily consists of partial mastectomy or excisional biopsy specimens. This study does not compare the classification performance of the ABN-DCN model on small biopsies versus larger excision specimens. Therefore, it is not possible to assess the diagnostic challenges faced by general pathologists on small biopsies versus larger excision specimens in this particular research.

### Comparison with state-of-the-art methods

There have been numerous studies on breast histopathology image classification using CNNs^16^^,^^17^^,^^19^, ^20^, ^21^, ^22^, ^23^^,^^33^^,^^43^ and these studies have achieved higher than 90% classification accuracy. Table 2 lists a sample of the openly accessible reported works on classification using BreaKHis dataset. On the BreaKHis database, the majority of the work showed classification performance higher than 90% accuracy.^16^, ^17^, ^18^^,^^21^^,^^22^^,^^37^^,^^43^^,^^69^^,^^70^ They haven't offered visualization of the region of interest where the model focuses for decision making while reporting greater classification performance. Recently, the authors attempted to highlight the regions of interest by fusion of visual explanation with CNN models.^71^^,^^72^ The propitious results serve as a true testimony to the propriety of the proposed approach with enhanced performance improvements in comparison to prevailing approaches.

**Table 2:** Performance comparison of ABN-DCN model with state-of-the-art results.

Paper	Methods	Accuracy
Spanhol et al^16^	AlexNet	85%
Bardou et al^17^	ensemble model and SVM	97%
Togacar et al^19^	BreastNet	98%
Xie et al^21^	Inception v3 and Inception ResNet v2	98%
Liew et al^22^	DenseNet, XGBoost	97%
Gupta et al^37^	DenseNet	95%
Thuy et al^43^	VGG16, VGG19	98%
Das et al^69^	VGG Net with Multiple Instance pooling layer	89.52%
Chattopadhyay et al^71^	Dense Residual Dual Shuffle Attention Network	98.1%
Lei et al^72^	Res2Net Based on Multi-scale Attention Mechanism	98.5%
Sharma et al^73^	Xception and SVM	96.25%
**Proposed ABN-DCN Model**	**ABN, Variant of DarkNet19**	**98.7%**

The outcome of this diagnostic study demonstrated the ability of DCN model for the classification of breast histopathology images. This study also showed how the DCN model's classification performance is improved when the attention branch is integrated with the DCN model to form the Attention Branch Network (ABN). The ABN-DCN model's classification performance on the BreaKHis dataset was in par or superior to state-of-the-art performances. These findings are important because the proposed ABN-DCN model reported better classification performance and provided diagnostic interpretability by addressing the visualization of the cancerous regions in the histopathology images where the model focuses on decision making.

The attention branch of ABN produces a heatmap that highlights the regions of interest and reveals the key features of the image relevant for model's decision. This allows us to gain insight into the model's decision-making process, which can be used to improve the model further or provide an explanation for the medical experts. The baseline DCN model, supplemented with the ABN approach, has not been reported in the published literature in the biomedical domain to develop an interpretable decision-support model for accurate cancer prediction appended by diagnostic interpretability.

## Concluding remarks and future plan

This paper put forth a novel interpretable decision-support model, outfitted with classification and visualization of histopathological images for the decisive detection of cancer. A convolutional neural network with supervision on the precise regions that offers explicit attention on the attention maps for the classification of breast cancer histopathological images was proposed in this study. Transfer learning technique was deployed, with a baseline DCN model as the feature extractor, for the classification of histopathological images. The diagnostic interpretability of DCN model was achieved by integration of attention branch with the feature extractor, crafting an innovative Attention Branch Network (ABN). The proposed method highlights diagnostically relevant regions in the histopathology images and utilized the predicted region to classify benign and malignant tumors in the classification branch. The model exactly locates epithelial areas in the images to correctly classify benign and carcinoma tumors. This proved that the produced feature activations in the proposed model are consilient with the pathologist's findings, making the model a promising solution for providing diagnostic interpretability.

The proposed model can be further explored to a large scope. Strengthened by the combined benefit of diagnostic interpretability on network decision and enhanced classification performance, the proposed model can be utilized to develop a reliable computer-aided diagnosis system to automatically detect breast cancer from histopathology images. The proposed ABN-DCN model can be fine-tuned on histopathology images with different magnification factors that can simultaneously analyze both architectural and cytomorphological details with subtler architectural features, such as the “stiffness” of structure to predict the breast cancer of each patient without the need for manually switching microscope magnification. The proposed work can be further extended to utilize the high-resolution Whole Slide Imaging (WSI) samples and precisely locate the region of interest to demonstrate its ability to handle substantial images derived from a single biopsy. Future work could include addressing: (i) the multi-class classification of samples, (ii) patient-level classification combining magnification-independent dataset. Additional studies utilizing other prominent CNN models and independent datasets containing intermediate borderline lesions and clear-cut biopsy cases can be pursued to further explore the correlation between deep learning-based extracted images and clinical diversity.

## Funding

This research did not receive any specific grant from funding agencies in the public, commercial, or not-for-profit sectors.

## Code Availability

The code is available at https://github.com/Sruthisanalkumar/ABN-DCN-Model

## Conflict of interests

The authors declare that they have no conflict of interests.

## Data Availability

The datasets analyzed during the current study are available in the Breast Cancer Histopathological Database (BreaKHis) https://web.inf.ufpr.br/vri/databases/breast-cancer-histopathological-database-breakhis.
